# An Analysis of Health Care Team Communication Needs Among Younger vs Older Breast Cancer Survivors: Web-Based Survey

**DOI:** 10.2196/31118

**Published:** 2022-03-18

**Authors:** Deborah Vollmer Dahlke, Aya Yoshikawa, Molly McAdam, Sharyn Malatok, Elaine D Gonzales

**Affiliations:** 1 Texas A&M Center for Population Health and Aging Texas A&M University Austin, TX United States; 2 Texas Woman's University School of Health Promotion and Kinesiology College of Health Sciences Denton, TX United States; 3 Breast Cancer Resource Center Austin, TX United States

**Keywords:** breast cancer, breast cancer survivorship, patient-physician communications, patient-centric communication, younger breast cancer patients, patient communication

## Abstract

**Background:**

Prior studies indicate that the age of onset of breast cancer is an important element in considering communication between patients and the health care team. Younger women aged 45 and under diagnosed with breast cancer are often at a higher risk of being more vulnerable to psychosocial issues compared to older women aged 46 years and above. Few studies have examined age differences in patient perceptions of treatment-related discussion and communication during transition with their health care team.

**Objective:**

The aims of this survey were (1) to better understand breast cancer survivors’ perspectives regarding communication with health care providers during treatment and during transition to posttreatment care; and (2) to determine the differences between younger women with breast cancer (≤45 years of age) and older women (≥46 years of age). It was hypothesized that (1) breast cancer survivors’ psychosocial and finance-related communications with health care providers may lack effectiveness; (2) younger women experience greater needs for patient-centered communication with physicians and health care providers, especially about psychosocial care and transition to posttreatment care; and (3) younger breast cancer patients (≤45 years of age) need more information on survivorship and follow-up care.

**Methods:**

An internet-based survey was conducted with 143 women in Central Texas with 35% (n=50) aged 45 years or under and 65% (n=93) aged 46 years and above. The Mann-Whitney *U* test was performed to assess differences in participants’ perceptions about communication with health care providers by age group: younger (≤45 years of age) and older (≥46 years of age) women.

**Results:**

Statistically significant results pertained to rating health care team and patient discussions about transition from treatment to posttreatment using scores of 0 as “no discussion” and 100 as “in-depth discussion.” For the questions about management of posttreatment care, the overall mean score of the groups was 56.26 and that of the younger group was 43.96; the mean score of the older group was 61.96 (*P*=.02). For the question about the timing of follow-up appointments, the overall mean score was 64.29; the mean score of the younger group was 54.44, and that of the older group was 68.88 (*P*=.05). All the group scores related to psychosocial and financial support discussions with health care providers were low, with a rollup average of only 30.02 out of 100, suggesting that this is an important area for improving patient-centered communication.

**Conclusions:**

For all patients, transition from treatment to posttreatment requires a greater level of engagement and communication with the health care team. It appears that younger patients aged ≤45 years require more in-depth and personalized messaging to better understand their posttreatment care requirements.

## Introduction

Although the median age at presentation is approximately 62 years in the United States [1], approximately 11% of all breast cancers occur in women younger than 45 years [2]. Breast cancer survivors diagnosed at younger ages are confronted with multiple demands of managing families and careers as well as complex medical, psychosocial, and behavioral late effects, including fertility and mental health issues. They may also be dealing with financial toxicity as a result of their treatment and may lack health benefits including sick leave and paid time off [3,4].

Compared to older women, younger women generally have more aggressive cancers, lower survival rates, and are more likely to experience recurrence of cancer [5-10]. Communication between patients and their health care teams is critical for the delivery of high-quality, patient-centered care, and it is associated with improved adherence to posttreatment protocols, patient satisfaction, and self-management [11,12]. Health care team members may include oncologists, nurses, social workers, and patient navigators.

In this study, we used an internet-based survey tool to better understand the differences in the perceptions on health care provider team support between younger and older breast cancer survivors during treatment and the transition from treatment to posttreatment care. We hypothesized that (1) there may be breakdowns in communication during treatment and transitions in care between breast cancer and health care providers and (2) that younger women (ie, aged ≤45 years) have greater needs for patient-centered communication, especially that related to psychosocial care and the transition to posttreatment care. An additional hypothesis was that younger breast cancer patients (<45 years of age) need more information on survivorship and follow-up care during transition, as they are expected to live longer and may also have a higher rate of recurrence [12].

## Methods

### Overview

The Breast Cancer Resource Center (BCRC) was the recipient of a 5-year cooperative agreement with the US Center for Disease Control (CDC) under a grant entitled “Multiple Approaches to Support Young Breast Cancer Survivors and Metastatic Breast Cancer Patients.”

The grant is focused on improving services and access to resources for young breast cancer survivors diagnosed under the age of 45 years and for metastatic breast cancer patients. A multifaceted needs assessment, consisting of focus groups, key informant interviews, and an internet-based survey, was conducted to determine what unmet needs exist in Central Texas.

Participants were able to access it via the internet from a link provided by BCRC to its constituents and collaborating organizations from August to November 2020. There were no incentives for participants who completed the survey. Results were screened for automated agents or bots and duplicate entries. BCRC sought institutional review board approval from the University of Texas at the Austin Office of Research Support and Compliance and was granted an exemption (reference: FWA # 00002030).

### Recruitment

The participants were recruited using convenience sampling via email from a number of Texas-based cancer and breast cancer advocacy groups. The Cancer Alliance of Texas was also involved in the recruitment process by distributing surveys among its participating organizations, agencies, institutions, and individual members. BCRC formed an advisory council, consisting of physicians, researchers, stakeholders, and survivors, to support the CDC grant activities, including assisting in dissemination of the survey. The survey was also advertised on the BCRC Facebook pages, and the link was emailed to anyone who had been a BCRC client since 2018. The web-based survey was a voluntary, open survey, and it was created and distributed by BCRC to breast cancer survivors in Texas who had a previous or current breast or metastatic breast cancer diagnosis.

### Survey Design

The survey consisted of 44 questions and was created using Qualtrics (Qualtrics International). Content from earlier focus groups and key informant interviews informed the questions included in the survey and the draft survey was tested with survivors and members of the project’s advisory group. Anonymous responses were captured directly in Qualtrics, and they were later downloaded directly from the software for analysis.

Survey participants were informed of the average length of time the survey would take, the purpose of the survey, how the responses would be used, and who the investigator was. Adaptive questioning was used to reduce the number and complexity of the questions. Survey participants could go back and review their responses before submitting. The survey collected various demographic data including ethnicity, education level, and income and insurance status. Women were asked to rate the helpfulness of their health care team, as it related to aspects of treatment. Participants were queried about their concerns regarding treatment-related issues and the level of discussion with their health care providers about treatment-related, and psychosocial- and finance-related topics using a scale ranging from 0 for “not at all a concern/no discussion” to 100 for “extreme concern/in-depth discussion.” Cronbach α values for concerns about treatment-related issues, the level of discussion about treatment-related topics, and the level of discussion about psychosocial- and finance-related topics were. 81, .95, and .97, respectively.

### Data Analysis

Descriptive statistics and chi-square tests were conducted to examine associations of the sociodemographic characteristics, marital status, and the length of time since initial treatment with the age group. Because of the relatively small sample size, the Mann-Whitney *U* test was performed to examine differences in participants’ perceptions about communication with health care providers by age group: younger (<45 years old) and older (≥46 years old) participants. When analyzing missing data, we confirmed that the variables of interest were missing completely at random (Little’s missing completely at random test, *P*>.05); thus, the results using the listwise deletion method were reported [13]. All statistical analyses were conducted with Stata 17.0 (StataCorp LLC).

## Results

A total of 143 participants completed the survey and provided their year of birth. Among those participants, 140 identified themselves as women and 2 as other. The median age of the participants was 49.0 years with 35% (n=50) aged <45 years and 65% (n=93) aged >46 years. The median age of the younger breast cancer participants was 41.5 years, and that of the older breast cancer participants was 56.0 years. Among all the respondents, 83.6% (n=117) were White with 16.4% (n=23) reporting “other.” Latino or Hispanic ethnicity was claimed by 17.0% (n=24). The differences in education levels between the groups were significant and generally high with 18.9% (n=27) having a high school degree or some college education; 48.3% (n=69) held associate or bachelor’s degrees, and 32.9% (n=47) mentioned advanced master’s, professional, or doctoral degrees. Approximately 78.3% (n=112) of the participants were insured through their employer or had private insurance at the time of their diagnosis. The characteristics of all the participants are presented in Table 1.

Among the set of questions about their concerns regarding treatment-related effects, only 2 responses showed statistically significant differences between the younger (<45 years of age) and the older (>46 years of age) participants, as observed in Table 2. The question about concerns regarding genetic counseling had a mean of 65.40 for all participants with a mean of 73.60 for the younger group and 60.28 for the older group (*P*=.04). The question about concerns related to fertility preservation was significant, with means of 25.60 for all the participants, 45.70 for the younger participants, and 4.59 for older participants (*P*=.002).

**Table 1:** Survey participant characteristics.

Characteristics		Total N=143	≤45 years n=50 (35%)	≥46 years n=93 (65%)	Group test *P* value	
Age in years, n, Mean (SD)		N/A^b^	50, 40.7 (4.5)	93, 57.6 (9.3)		
**Gender, n (%)**						N/A
	Female	140 (97.9)	50 (100)	90 (96.8)		
	Other	2 (1.4)	0 (0)	2 (2.2)		
	Missing	1 (0.7)	0 (0)	1 (1.1)		
**Race, n (%)**						.14
	White	116 (81.1)	37 (74)	79 (84.9)		
	Other	23 (16.1)	11 (2)	12 (12.9)		
	Missing	4 (2.8)	2 (4)	2 (2.2)		
**Ethnicity, n (%)**						.49
	Spanish, Hispanic, or Latino	24 (16.8)	10 (20)	14 (15.1)		
	Other	117 (81.8)	40 (80)	77 (82.8)		
	Missing	2 (1.4)	0 (0)	2 (2.2)		
**Education, n (%)**						*.05*
	High school graduate or some college with no degree	27 (18.9)	12 (24)	15 (16.1)		
	Associate's or bachelor’s degree	68 (47.6)	28 (56)	40 (43)		
	Master's, professional, or doctoral degree^a^	47 (32.7)	10 (20)	37 (39.8)		
	Missing	1 (0.7)	0 (0)	1 (1.1)		
**Income in US $, n (%)**						.48
	<$10,000 to $39,999	26 (18.2)	12 (24)	14 (15.1)		
	$40,000 to $79,999	40 (28)	14 (28)	26 (28)		
	$80,000 to ≥ $150,000	73 (51)	24 (48)	49 (52.7)		
	Missing	4 (2.8)	0 (0)	4 (4.3)		
**Marital status, n (%)**						.79
	Married	94 (65.7)	33 (66)	61 (65.6)		
	Not married/other	48 (33.6)	17 (34)	31 (33.3)		
	Missing	1 (0.7)	0 (0)	1 (1.1)		
**Length of time since initial treatment, n (%)**						.37
	Still in active treatment	21 (14.7)	10 (20)	11 (11.8)		
	Less than 6 months	14 (9.8)	5 (10)	9 (9.7)		
	1-4 years	51 (35.7)	18 (36)	33 (35.5)		
	More than 5 years	26 (18.2)	6 (12)	20 (21.5)		
	Missing	31 (21.7)	11 (22)	20 (21.5)		
**Insurance status, n (%)**						N/A
	Insured through employer or private insurance purchased	112 (78.3)	43 (86)	69 (47.2)		
	Medicare or secondary insurance	14 (9.8)	1 (2)	13 (14)		
	Medicaid for breast and cervical cancer or Medicaid	3 (2.1)	1 (2)	2 (2.2)		
	Military (TRICARE)	4 (2.8)	2 (4)	2 (2.2)		
	Not insured	10 (7)	3 (6)	7 (7.5)		

^a^Professional or doctoral degrees: Juris Doctor and Doctor of Medicine.

^b^N/A: Not applicable. Chi-square tests were not possible due to insufficient observations for this category.

**Table 2:** Mann-Whitney *U* test results regarding concerns about treatment-related issues (N=143).

From the following list of treatment-related issues, please rate each of the following concerns (0=Does/did not at all concern you; 100=Extreme concern)	Total				≤45 years old				≥46 years old				Group test *P* value^a^
	n	Mean (SD)	Median	n		Mean (SD)	Median	n		Mean (SD)	Median	N/A	
Need for genetic counseling	104	65.40 (32.47)	71.00	40		73.60 (30.43)	82.50	64		60.28 (32.88)	59.50	*.04*	
Access to fertility preservation	45	25.60 (37.76)	3.00	23		45.70 (43.75)	30.00	22		4.59 (8.77)	1.00	*.002*	
Chemotherapy side effects	99	76.32 (29.61)	90.00	39		77.54 (27.69)	82.00	60		75.53 (30.99)	90.00	.77	
Mastectomy	109	71.94 (31.78)	80.00	43		77.65 (28.47)	98.00	66		68.23 (33.45)	76.50	.09	
Managing all my prescribed medications and treatments	98	64.70 (30.20)	68.00	40		65.05 (24.76)	64.50	58		64.47 (33.66)	72.50	.73	
Hair Loss	90	67.08 (32.03)	74.00	37		67.97 (31.68)	75.00	53		66.45 (32.56)	73.00	83	
Managing pain and discomfort	104	72.08 (25.05)	76.00	39		75.49 (22.20)	80.00	65		70.03 (26.57)	74.50	.33	
Reconstructive surgery	97	74.43 (29.55)	76.00	40		82.23 (21.23)	90.00	57		68.96 (33.30)	78.00	.07	
Using medications to manage long-term side effects	97	71.14 (29.06)	85.00	35		69.60 (28.43)	76.00	62		72.02 (29.61)	80.00	.56	
Managing ongoing side effects of treatment	107	76.67 (24.97)	84.00	41		76.54 (21.21)	80.00	66		76.76 (27.20)	85.00	.58	
Finding undergarments/clothes/wigs to wear after surgery/treatment	93	57.67 (31.71)	60.00	35		57.03 (32.35)	65.00	58		58.05 (31.60)	59.50	.87	
Average	N/A^b^	65.73	69.73	N/A		69.85	74.82	N/A		62.31	68.14	N/A	
Rollup	N/A	67.99	75.00	N/A		71.06	79.00	N/A		65.98	75.00	N/A	

^a^Italicized *P* values are statistically significant.

^b^N/A: not applicable.

The next set of questions focused on transitions to posttreatment care. This was the area where we found the greatest number of significant differences between the younger (<45 years of age) and older participants (>46 years of age), and where the second hypothesis that younger breast cancer survivors experience less support from their health care teams appeared to be best demonstrated. The questions asking about transitions to posttreatment care were ranked based on the extent to which treatment-related topics were discussed by the health care team during the transition to posttreatment care on a scale from 0 indicating “no discussion” to 100 indicating “in-depth discussion.” Table 3 provides the results from this set of questions addressing our second hypothesis suggesting that there are areas of communication breakdown or lack of communication between breast cancer survivors and their health care team during transition to posttreatment care. There are “rollups” of the scores in Table 3 and Table 4 that provide a summary of the preaggregated values for the mean and median.

**Table 3:** Mann-Whitney *U* test results for posttreatment-related topics (N=143).

To what extent did your health care team discuss the following treatment-related topics with you during your transition from treatment to posttreatment care (0=No discussion; 100=In-depth discussion)?	Total				≤45 years old				≥46 years old				Group test *P* value^a^
	n	Mean (SD)	Median	n		Mean (SD)	Median	n		Mean (SD)	Median	N/A^b^	
Which doctor would manage your posttreatment care?	82	56.26 (32.17)	52.00	26		43.96 (32.22)	36.00	56		61.96 (30.78)	59.50	*.02*	
When to contact your oncologist vs your primary care doc vs your OB-GYN^c^?	72	46.10 (36.66)	35.00	22		32.77 (37.39)	13.00	50		51.96 (35.12)	50.00	*.03*	
What long-term effects of treatment to expect (eg, early menopause)?	77	55.77 (32.31)	60.00	27		43.52 (31.62)	50.00	50		62.38 (31.00)	63.50	*.02*	
What should you do for exercise and nutrition?	74	44.45 (29.51)	39.50	23		40.57 (30.18)	35.00	51		46.20 (29.33)	43.00	.45	
How frequently you should have follow-up appointments?	85	64.29 (31.23)	68.00	27		54.44 (33.45)	50.00	58		68.88 (29.31)	72.50	*.05*	
How often you would need scans/tests?	78	58.35 (31.61)	52.50	24		48.00 (29.00)	48.00	54		62.94 (31.89)	60.50	.06	
What are your chances for recurrence/metastatic breast cancer?	82	55.71 (31.84)	55.00	25		47.12 (30.99)	50.00	57		59.47 (31.74)	59.00	.11	
What symptoms should you look for recurrence or metastatic breast cancer?	76	50.13 (35.12)	49.50	22		44.10 (34.21)	37.00	54		52.59 (35.50)	51.50	.36	
What are your risks for other cancers?	67	47.19 (35.58)	39.00	22		42.27 (37.89)	31.50	45		49.60 (34.58)	49.00	.36	
Your survivorship and treatment care plan or next step summary	67	55.91 (34.88)	53.00	19		43.05 (35.73)	45.00	48		61.00 (33.56)	62.50	.06	
Average	N/A	53.41	50.35	N/A		53.41	50.35	N/A		57.70	57.10	N/A	
Rollup	N/A	53.71	51.00	N/A		44.26	40.00	N/A		57.99	55.00	N/A	

^a^Italicized *P* values are statistically significant.

^b^N/A: not applicable.

^c^OB-GYN: obstetrician-gynecologist.

The statistically significant questions where the younger participants responded with lower scores than the older breast cancer survivor participants regarding the extent to which their health care provider discussed transition topics included the following:

- Which doctor (oncologist vs primary care) would manage posttreatment care? The younger participants’ mean score was 43.96 vs the older participants’ mean score of 61.96 (*P*=.02).
- When to contact your oncologist or primary care doctor or your OB-GYN (obstetrician-gynecologist)? The younger participants’ mean score was 32.77, and the older participants’ mean score was 51.96 (*P*=.03).
- What long-term effects to anticipate (ie, early menopause)? The younger participants’ mean score was 43.52, and that of the older participants was 62.38 (*P*=.02).
- How frequently you should have follow-up appointments? The younger participants’ mean score was 54.44, and that of the older participants was 68.88 (*P*=.05).

These results address our second hypothesis and suggest that younger women may have greater needs for patient-centered communication with physicians and health care providers, especially for psychosocial care and during transition to posttreatment care. The results also address our third hypothesis that younger breast cancer survivors need patient-centered communication and information on survivorship and follow-up care during transitions to posttreatment care.

Among the other nonstatistically significant questions, there were several that showed large differences between the 2 means, indicating that these questions too may be important differentiators between the needs of younger and older breast cancer survivors. These included questions about the need for scans/tests or summaries of next treatment steps. Overall, the group mean scores regarding transitions in care were relatively low with an average rollup score of 53.71 for all the questions regarding this stage of care.

Although there were no statistically significant differences in the levels of discussions with health care teams about psychosocial- and finance-related topics between the younger and older participants, the overall scores for the group based on the extent of the discussions with health care providers were all less than 50, ranging from a low mean of 22.54 for concerns about remaining medical bills to a high mean of only 34.53 for questions about the need for financial service counseling or support. The rollup of the means and medians for this area of questioning was 30.02. Table 4 shows the means and medians of this group of questions for the entire participant group and for the younger and older groups as well as the average and rollup scores.

**Table 4:** Scores related to psychosocial and finance-related discussions with health care providers.

To what extent did your health care team discuss the following psychosocial and finance-related topics with you during your transition from treatment to posttreatment care (0=No discussion; 100=In-depth discussion)?	Total				≤45 years old				≥46 years old				Group test *P* value
	n	Mean (SD)	Median	n		Mean (SD)	Median	n		Mean (SD)	Median	N/A^a^	
Your concerns about remaining medical bills	48	27.02 (30.65)	16.00	14		18.57 (27.31)	4.00	34		30.50 (31.64)	19.00	.22	
Your concerns about cost for posttreatment therapies and medication	44	33.52 (33.01)	28.00	15		21.47 (26.97)	9.00	29		39.76 (34.51)	37.00	.08	
Your need for financial service counseling or support	40	34.53 (36.78)	17.00	13		23.15 (27.48)	8.00	27		40.00 (39.81)	20.00	.32	
Your need for ongoing emotional/mental support or counseling	48	31.56 (29.75)	26.50	13		23.08 (26.88)	12.00	35		34.71 (30.51)	28.00	.31	
Supporting your spouse, children, and family members through posttreatment	32	28.28 (35.41)	8.00	10		15.30 (31.11)	2.00	22		34.18 (36.33)	17.50	.10	
Supporting your spouse, children, and family members through a diagnosis of metastatic breast cancer	28	22.54 (31.70)	5.00	9		7.22 (12.74)	0.00	19		29.79 (35.53)	18.00	.06	
Average	N/A	29.57	16.75	N/A		18.13	5.83	N/A		34.82	23.25	**N/A**	
Rollup	N/A	30.02	17.50	N/A		18.93	5.00	N/A		34.96	25.00	N/A	

^a^N/A: not applicable.

## Discussion

### Principal Findings

Our survey results demonstrated that breast cancer survivors experience barriers or gaps in communication with their health care teams during transition from treatment to posttreatment care. We observed that younger breast cancer survivors have lower statistically significant scores regarding the depth of communications with health care providers pertaining to transitions to posttreatment regarding when to contact which care provider (ie, oncology team vs primary care), what long-term effects to anticipate, and how often they would need follow-up scans or tests. For younger and older participants, the mean scores for what would be considered critically important aspects of cancer survivorship fell below 60 points on the 100-point scale of 0 for “no discussion” and 100 for “in-depth discussion.”

For the questions in the areas of communications with health care team members regarding psychosocial and finance-related topics, our results were comparable to the findings in a nationally representative sample in which limited proportions of cancer survivors reported high-quality discussions with providers after diagnosis, ranging from 29% (n=349) for emotional and social needs to 62% (n=745) for follow-up care recommendations, indicating that 76% experienced suboptimal communication with their cancer care providers [14].  These relatively low scores for patient-provider communication are concerning, especially the apparent lack of discussion about the late and long-term effects of treatment. A number of studies have shown that cancer survivors face many challenging physical and psychological effects of treatment that fundamentally shape their quality of life [14,15]. This concern is well documented, especially for younger survivors [16-23]. Research in this area strongly supports the need for improvement in patient-focused communication among providers and oncology health care team members.

Regarding younger breast cancer survivors as compared to older survivors, patient-specific communication assumes additional importance, as shown by the findings of Champion et al [24]. This work was a retrospective study involving more than 500 breast cancer survivors aged 25 to 50 years, showing that women experienced long-term difficulties with emotional and social functioning, which increased with decreasing age at diagnosis. In their study, younger breast cancer survivors experienced lower vitality and higher rates of depression in comparison to age-matched healthy controls and women who were older at diagnosis. The conclusions drawn by Champion et al suggested that women diagnosed with breast cancer at a younger age (<45) are at significant risk for emotional and psychosocial sequelae during and after breast cancer treatment. Their research suggested that younger women require age-specific psychosocial support, ideally in the context of coordinated multidisciplinary care teams [24,25].

This need for support is further supported by the study of Johnson et al addressing breast cancer in adolescents and young adults [26]. In this study, the researchers found that concerns about fertility, sexuality, body image, and disruptions in peer and romantic relationships as well as financial and occupational difficulties and fear of death from cancer are more pronounced in younger breast cancer survivors than in older survivors, and that these concerns my contribute to survivor distress [26].

Our study was cross-sectional, thus limiting the ability to draw causal inferences. We could not control for certain variables, such as the cancer site, stage or subtype, provider type, or specialty due to sample size limitations and lack of information. The mix of younger (≤45 years of age) and older (≥46 years of age) participants in our survey, with 35% being younger, is higher than the national ratio of 11% younger (≤45 years of age) patients [3]. This could affect the group means and the rollup scores. This age group is also primarily reflective of Central Texas and especially the Travis County catchment area in which the median age is 34.2 years [27]. However, we may have had responses from other areas of Texas, and thus our survey is not necessarily representative of Central Texas or Texas in general. Our sample was small, partially due to missing data; therefore, this limited our analysis to determining differences in participants’ perceptions about communication with health care providers by age group. We confirmed that the missing data met the assumption of MCAR (Little’s missing completely at random test, *P*>.05) and employed the listwise deletion method, a common method to generate unbiased and conservative estimates [16].

The survivors’ cancer history was self-reported. Our sample was predominantly composed of non-Hispanic Whites and communication differences may exist among patients from diverse racial, ethnic, and cultural backgrounds. There was also the possibility of recall bias, particularly for respondents further from treatment. Our study was conducted during the period of sequester in Central Texas due to the COVID-19 pandemic, which may account for a slightly lower response rate.

### Conclusions

Breast cancer survivors’ perceptions of conversations with health care professionals revealed missed opportunities for older and younger patients regarding understanding of concerns related to costs, the need for financial services, emotional/mental support counseling, and the need for providing patients’ spouses and children with posttreatment support. Participants in this survey emphasized additional support for spouses, children, and family members of those diagnosed with metastatic breast cancer. This research also revealed missed opportunities for enhancing patient-provider communication among younger breast cancer patients during treatment regarding genetic screening and fertility preservation services.

Younger and older breast cancer survivors transitioning from treatment to posttreatment care would benefit from being offered access to psychosocial and financial counseling following breast cancer treatment. These gaps and barriers imply the need for oncology care teams to increase their focus on communications and clarity regarding transitions in care, follow-up care, late or long-term treatment effects, financial support, and psychosocial needs, with special focus on younger breast cancer patients.

## Abbreviations

BCRC: Breast Cancer Resource Center
